# An association of a simultaneous nuclear and cytoplasmic localization of Fra-1 with breast malignancy

**DOI:** 10.1186/1471-2407-6-298

**Published:** 2006-12-28

**Authors:** Yuhua Song, Santai Song, Dong Zhang, Yan Zhang, Liyong Chen, Lu Qian, Ming Shi, Huibin Zhao, Zefei Jiang, Ning Guo

**Affiliations:** 1 Institute of Basic Medical Sciences, Beijing 100850, P.R. China; 2 307 Hospital, No. 8 East Street, Fengtai District, Beijing 100071, P.R. China; 3 The Third Military Medical University, Chongqing 400038, P.R. China; 4 The department of Oncology, Affiliated Hospital of Qingdao University, Qingdao, Shandong 266003, P.R. China

## Abstract

**Background:**

Overexpression of Fra-1 in fibroblasts causes anchorage-independent cell growth and oncogenic transformation. A high level of Fra-1 expression is found in various tumors and tumorigenic cell lines, suggesting that Fra-1 may be involved in malignant progression. This study aimed to investigate the significance of Fra-1 expression in breast carcinogenesis.

**Methods:**

The expression of Fra-1 was investigated by immunohistochemistry in neoplastic breast diseases ranging from benign fibroadenoma to very aggressive undifferentiated carcinoma. The correlations of Fra-1 expression with other indicators of breast carcinoma prognosis (ER, PR and ErbB2 receptors) were analyzed.

**Results:**

All neoplastic breast tissues, either benign or malignant breast tissues, were nuclear immunoreactive for Fra-1-recognizing antibody. The pattern of Fra-1 expression by benign neoplastic cells was predominantly nuclear. However, the nuclear/cytoplasmic concomitant immunoreactivity was observed in all types of breast carcinomas. A clear shift in Fra-1 immunoreactivity, from an exclusively nuclear to a simultaneous nuclear and cytoplasmic localization was noticed in ~90% of breast carcinomas.

**Conclusion:**

The overall expression, pattern and intensity of Fra-1 proteins were correlated with breast oncogenesis. Overexpression of Fra-1, leading to a persistent high cytoplasmic accumulation, may play a role in the process of breast carcinogenesis.

## Background

Transcription factor AP-1 (activator protein 1) is thought to play an important role in regulating the gene expression pattern in response to external stimuli [1-4]. It is composed of transcription factors belonging to Jun and Fos families [5,6]. In mammalian cells, three members of the Jun family (c-Jun, JunB, and JunD) and four members of the Fos family (c-Fos, FosB, Fra-1, Fra-2) have been identified to date. These two family members can form Jun/Jun homodimers or more stable Fos/Jun heterodimers, which bind to the palindromic TGA(C/G)TCA recognition sequence, so-called 12-O-tetradecanoylphorbol-13-acetate (TPA) response element (TRE) and activate the gene transcription [7-9]. Since TRE-containing promoter constructs were strongly activated by the tumor promoter TPA and AP-1 proteins (c-Jun and c-Fos) [10], the AP-1 complex was implicated in carcinogenesis soon after discovery.

In eukaryotic cells, extracellular stimuli cause a series of intracellular signals that ultimately activate the proteins binding to transcriptional control elements, selectively regulating gene expression. Fos and Jun family members participate at the end of signal transduction pathways [11-14]. Stimulation with various growth factors and activated oncogenes results in immediate transcriptional activation of the c-Fos gene, accompanied with transient overexpression of c-Fos protein and formation of c-Jun/c-Fos heterodimers [15-17]. Whereas, Fra-1 expression is delayed and more stable. In exponentially growing cells, Fra-1 protein is hyperphosphorylated and its expression elevated [18].

All AP-1 proteins are characterized by a basic leucine-zipper region. As the transcription factors, c-Fos and FosB proteins harbor a C-terminal transactivation domain, but Fra-1 and Fra-2 lack this region [19,20]. Since Fra-1 and Fra-2 were shown to inhibit c-Fos- and c-Jun-dependent transactivation in a transient-transfection assay, it has been proposed that these proteins act as negative regulators, which limit the duration of the AP-1 response [21]. However, emerging evidence suggests an important role for Fra-1 in cell motility, invasion, and progression of the transformed state in several cell types [22-24]. The recent data indicated that overexpressed Fra-1 in fibroblasts causes anchorage-independent growth and oncogenic transformation [10,25]. A high level of Fra-1 expression is found in some tumors and tumorigenic cell lines [26-30]. These results suggested that Fra-1 might be involved in malignant progression. However, few reports deal with the expression and location of Fra-1 protein in human breast tumor tissues.

In this study, we conducted retrospective study using 61 paraffin-embedded breast tumor tissues to investigate Fra-1 expression by immunohistochemistry. The correlations of Fra-1 with estrogen receptor (ER), progesterone receptor (PR) and ErbB2 receptor status were analyzed in breast cancer patients in order to further explore the role of Fra-1 in the diagnosis of breast cancer.

## Methods

### Collection of Breast Tissue Samples

A total of 61 breast tissue specimens constituted by fibroadenomas, hyperplastic adenosis/fibroadenomas, ductal carcinomas, lobular carcinomas and other breast carcinomas were collected at 307 Hospital (Beijing, China) from patients undergoing surgery (Table 1). All tumor tissue samples were fixed immediately after surgical removal in 10% buffered formalin and embedded in paraffin.

### Immunohistochemistry

Paraffin-embedded tissue was freshly cut (4 μm). The sections were dewaxed with xylene, and gradually hydrated in a decreasing ethanol series ending in distilled water. Endogenous peroxidase activity was quenched using 3% hydrogen peroxide in distilled water for 5–10 minutes and then washed in phosphate buffered saline (PBS) before immunohistochemical staining. The Streptavidin-Biotin method was used to detect Fra-1 expression. Antigen retrieval was achieved using microwave heating in 1 mM EDTA (pH 9.0) at ~97°C for 20 minutes, followed by a 20 minute cooldown period at room temperature. The rabbit polyclonal antibody against the synthetic peptide derived from residues 128–180 of human Fra-1 (ab22837, Abcam Lid. UK) at a concentration of 5 μg/ml in PBS was applied for 60 minutes at 37°C or overnight at 4°C. The antibody is not able to detect the other members of Fos family. After washing with PBS, sections were incubated with biotinylated anti-rabbit antibody (Zhongshan Co., Beijing, China) at 37°C for 15 minutes and washed in PBS. Then the streptavidin peroxidase reagent (Zhongshan Co., Beijing, China) was applied at 37°C for 15 minutes and sections were again washed in PBS. The color was developed by a 7-minute incubation with 3,3'-diaminobenzidine (DAB) solution and the sections were weakly counterstained with hematoxylin, washed, dehydrated with alcohol and xylene and mounted with coverslips. Omission of the primary antibody and substitution by nonspecific immunoglobulin at the same concentration were used as negative controls. For ER, PR, and ErbB2 staining, sections were stained with the ER/PR/ErbB2 Immunohistochemistry Test Kit (Zhongshan Co., Beijing, China), which includes the mouse monoclonal antibodies against human ER, PR, and ErbB2 proteins.

### Assessment of Fra-1, ER, PR and ErbB2 expression

Fra-1 expression is purely nuclear or mixed nuclear and cytoplasmic. Nuclear reactivity was scored as high (>75% cell positive) or low (<75% cell positive). Cytoplasmic reactivity, when present (positive) was scored as high (strong staining) and low (weak staining). The percentage of tumor cells showing nuclear or cytoplasmic Fra-1 reactivity was recorded semi-quantitatively at ×400 magnification after examining the entire histologic section. ER and PR staining was scored in a minimum of 300 histologically identified neoplastic cells, showing nuclear reaction. Tumors with positive ER or PR nuclear staining in >10% of tumor cells were defined as ER or PR positive, respectively. For ErbB2 protein, immunostainings were scored as strong (2++ and 3+++), weak or negative (0 and 1+) according to the rate of labelled tumor cells and the membrane staining intensity. The ErbB2 status was further classified as overexpressed including 2++ and 3+++ (positive) or not significantly expressed (negative).

### Statistical analysis

Data were analyzed using standard statistical software SPSS version 13.0 (Chicago, Illinois) as previously described. Fisher's Exact Test was used to determine the significance of the association of the different factors. Expression is defined as negative or positive staining and staining pattern is defined as nuclear or cytoplasmic staining. *P *< 0.05 was considered significant.

## Results

**Figure 1:**
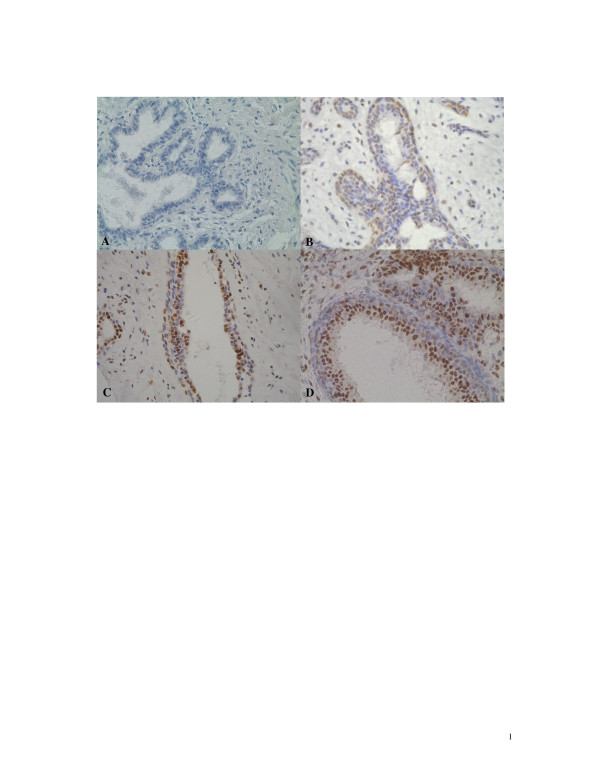
The immunohistochemical staining of Fra-1 in benign breast tumors. Paraffin-embedded tissue sections were prepared. Endogenous peroxidase activity was quenched using 3% hydrogen peroxide before immunohistochemical staining. The Streptavidin-Biotin method was used to detect Fra-1 expression. The rabbit anti-human Fra-1 polyclonal antibody at a concentration of 5 μg/ml in PBS was applied for 60 minutes at 37°C or overnight at 4°C. The sections were then incubated with biotinylated anti-rabbit antibody at 37°C for 15 minutes. The streptavidin peroxidase reagent was applied at 37°C for 15 minutes. The color was developed by incubating with DAB solution. A, nonspecific immunoglobulin was used as a negative control. B, The nuclear immunoreactivity for Fra-1 was identified mostly in the epithelial cells of fibroadenomas. C, The strong nuclear immunostaining was present in fibroadenomas. D, Positive staining appeared largely on the apical epithelial layer on the luminal aspect, but myoepithelial layer was mostly negative. ×400 magnification.

The results of the immunohistochemical study of 61 breast tissues (10 fibroadenomas, 10 hyperplastic adenosis/fibroadenomas, 31 ductal carcinomas, 4 lobular carcinomas and 6 other types of breast carcinomas) are summarized in Table 2. No staining was observed in the absence of the primary antibodies or with nonspecific immunoglobulin (Fig. 1A). Nuclear expression of Fra-1 was detected in all neoplastic tissues. In 85% of benign tumors (17/20), the immunoreactivity for Fra-1-recognizing antibody was exclusively restricted to the nuclei. Fig. 1B shows that the nuclear immunoreactivity for Fra-1 was identified mostly in the epithelial cells. Generally, the nuclei of infiltrating inflammatory cells were not stained. Moreover, the nuclear staining was not observed in all of the epithelial cells. Fig. 1C and 1D shows the strong immunostaining in fibroadenomas. In some ducts lined with stratified epithelium, positive staining appeared largely on the apical epithelial layer on the luminal aspect as shown in Fig. 1D, but myoepithelial layer adjacent to the acinal basement membrane was mostly negative. However, the weak nuclear staining of some interlobular stroma cells was also visible. In most cases (85%), the immunoreactivity is present only in the nuclei. In three cases (15%), focal unequivocal cytoplasmic staining was also exhibited.

**Figure 2:**
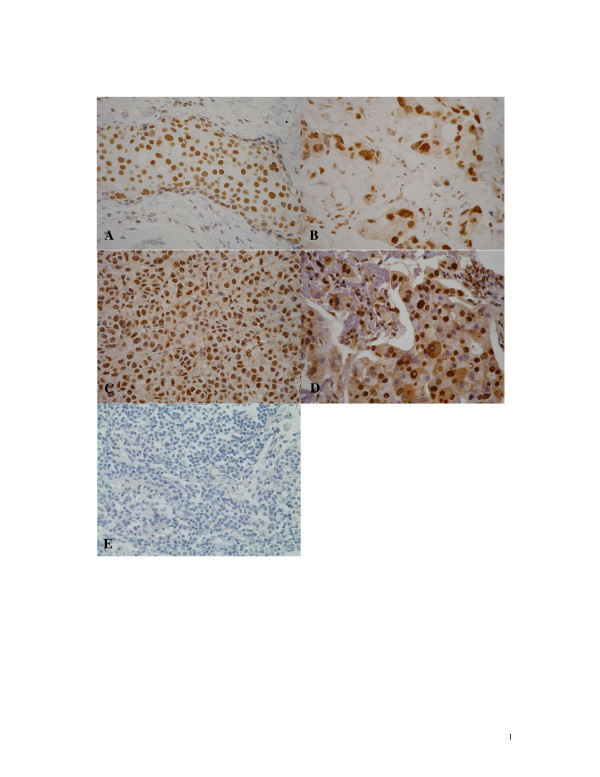
The immunohistochemical staining of Fra-1 in breast carcinomas. A and B, strong nuclear and weak cytoplastic immunostaining were observed in two representative invasive ductal carcinomas. C and D, Strong nuclear and cytoplasmic double staining was very pronounced in two representative invasive ductal carcinomas. E, nonspecific immunoglobulin was used as a negative control. ×400 magnification.

In contrast to fibroadenomas or hyperplastic adenosis/fibroadenomas, strong positive nuclear staining for Fra-1 was easily seen in all types of breast carcinomas, either diffuse (100% of the tumor cells positive) or focal (>75% of the tumor cells positive). Moreover, a variable degree of cytoplasmic staining was easily observed in ~90% breast carcinomas, although the immunoreactivity in cytoplasm was weaker than in nucleus. However, cytoplasmic localization of Fra-1 was rarely observed in adjacent peritumoural tissues. In normal epidermal tissues a weak immunoreactivity was only restricted to the nucleus of basal cell layer. In 56% of cases in breast carcinomas (23/41), weak cytoplasmic immunostaining was observed (Fig. 2A and 2B). It is noteworthy that in 34% cases (14/41), strong nuclear and cytoplasmic double staining was very pronounced (Fig. 2C and 2D).

**Figure 3:**
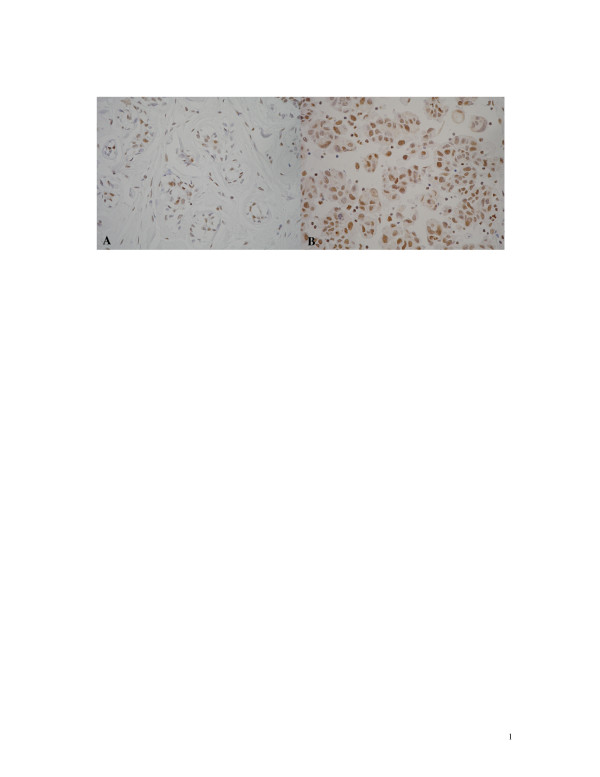
The differential expression of Fra-1 protein in breast carcinomas. Fra-1 protein immunostaining was weak in adjacent peritumoural tissues and positive reactivity restricted to nuclear (A), but strong in poorer differentiated region in the same section and positive reactivity presented in nuclear and cytoplasmic simultaneously (B). ×400 magnification.

The accumulation of Fra-1 protein and the changes occurring in its subcellular localization are illustrated in Figure 3A and 3B. Fra-1 protein immunostaining was usually weak in normal tissues or well-differentiated adjacent peritumoural tissues and positive reactivity restricted to the nucleus (Figure 3A). Whereas in poorer differentiated region in the same section the expression of Fra-1 protein was present in the nucleus and cytoplasm simultaneously (Figure 3B). In addition, Fra-1 expression was observed in all types of breast carcinomas.

The results provided in Table 3 showed an association between the cytoplasmic expression of Fra-1 and breast carcinoma. There was a significant relationship between the cytoplasmic expression of Fra-1 and breast carcinomas (*P *= 0.000). No inverse relationship between Fra-1 and ER and PR protein levels was noticed in malignant tumors (Table 4). The relative expression level of Fra-1 was not correlated with the expression of ErbB2.

## Discussion

A large group of oncogenes encode transcription factors. The oncogenic transcription factors play critical role in contributing to malignancy by altering programs of cell growth, differentiation and development. Activation of AP-1 has been implicated in the fundamental processes occurring in mammalian cells. Promoters and enhancers of many genes, whose expression is affected in cancer development, bear TRE capable of binding the Fos and Jun transcription factors. Recent reports have underlined a crucial role of Fra-1 in the transformation of several cell lines. For example, ectopic expression of Fra-1 in rat fibroblast cells induced tumorigenicity [25]. Fra-1 gene induction was required for establishment of the neoplastic phenotype in retrovirally transformed rat thyroid cells [31]. The overexpression of Fra-1 protein had also been described in a variety of epithelial neoplasias, including thyroid cancer [27], esophageal squamous carcinoma [32], endometrial cancer [33] and prostate cancer [34] A tight association of Fra-1 expression with highly invasive breast cancer cell lines has been demonstrated. Fra-1 was efficient for stimulating MMP-9, MMP-1, VEGF and cyclin D1 production in MCF7 cells [23] and could induce the expression of osteopontin (OPN), thrombospondin and CD44 [35], suggesting that Fra-1 might influence cell proliferation, invasion and angiogenesis. The effects of Fra-1 on motility and invasion have been observed in four highly invasive and nine weakly invasive human breast cancer cell lines by cDNA array technology. Among 24 differentially expressed genes, Fra-1 expression level was significantly enhanced in the highly invasive cells. Similar results were found by cDNA array technology in 22 human mammary epithelial cell strains/lines (breast cancer cell lines and cells derived from primary or metastatic breast cancers and reduction mammoplasties) [26]. Some authors suggested that Fra-1 might be a valuable diagnostic marker in breast cancer.

Fra-1 Expression at both mRNA and protein levels in breast cancer tissues has been previously studied using Northern blot and Western blot [36,37]. To further investigate the significance of Fra-1 in human breast cancer, the expression of Fra-1 was investigated in this study by immunohistochemistry with the anti-Fra-1 antibody in neoplastic breast diseases ranging from benign fibroadenoma to very aggressive undifferentiated carcinoma. The correlations of Fra-1 expression with other indicators of breast carcinoma prognosis (ER, PR and ErbB2 receptors) were analyzed. All neoplastic breast tissues, either benign or malignant breast tissues, were nuclear immunoreactive for Fra-1-recognizing antibody, whereas tumor adjacent normal tissues showed a much weaker nuclear immunoreactivity only restricted to some epithelial cells. The patterns of Fra-1 expression by benign neoplastic cells were predominantly nuclear. In only 3 of 20 cases of fibroadenomas or hyperplastic adenosis/fibroadenomas, nuclear expression coexisted with weak cytoplasmic reactivity. However, the nuclear/cytoplasmic concomitant immunoreactivity was observed in all types of breast carcinomas. A clear shift in Fra-1 immunoreactivity, from an exclusively nuclear to a simultaneous nuclear and cytoplasmic localization was noticed in ~90% of breast carcinomas. We can not exclude a slight increase in Fra-1 expression not detectable by immunohistochemistry. The data indicate that the expression pattern and intensity of Fra-1 proteins are correlated with epithelial cell oncogenesis.

A negative correlation of ER/PR and Fra-1 expression was observed in breast cancer cell lines. In breast cancer tissues, Fra-1 was found correlated with ER status in one study [37]. The high Fra-1 expression and phosphorylation in ER-negative cells appeared to take place only in culture conditions, since the phosphorylated bands were undetectable in clinical tumor tissue from both ER-positive and ER-negative carcinomas [37]. In another study, an inverse correlation of Fra-1 level with a progesterone receptor isoform PR-B expression was observed [38]. However, the correlations of Fra-1 expression with ER, PR and ErbB2 were not found in our study. The further investigation is still needed.

Fra-1 lacks a transcriptional transactivation domain and it is thought, in partnership with c-Jun, to drive the expression of genes [35]. Its overexpression results in the transcription of progression-associated genes and induction of epithelial mesenchymal transition [22,25]. The recent evidence has indicated a potential role for Fra-1 in abnormal differentiation and transformation of lung cells [19,39]. However, the role played by Fra-1 in malignancy remains unclear. It is known that many transcription factors shuttle between the nucleus and cytoplasm. The synthesis of Fra-1 is supposed to start in the cytoplasm. However when it was overexpressed, nuclear import might be hampered. Saturation of a nuclear import mechanism, leading to a persistent high cytoplasmic concentration of the proteins, may also play a role in the process of breast carcinogenesis.

The recent study showed that nuclear p21Cip1/WAF1 translocates to the cytoplasm and this translocation event is accompanied by resistance to various apoptotic stimuli [40]. The non-transcriptional mechanism of Fra-1 remains to be further demonstrated.

## Conclusion

In summary, our data by immunohistochemical analysis of breast tissues, including benign and malignant breast carcinomas, reveal that Fra-1 protein accumulation in the cytoplasm may play an important role in human breast carcinogenesis. These data may highlight the significance of therapy based on the blockage of Fra-1 functions of breast cancers.

## Competing interests

The author(s) declare that they have no competing interests.

## Authors' contributions

YS collected clinical data, carried out immunohistochemical study, performed statistical analysis and participated in writing the manuscript.

SS, ZJ recruited patients, designed and coordinated the study.

DZ, HZ collected clinical specimens.

YZ participated in the immunohistochemical studies

LY, LQ, MS evaluated the immunohistochemical data.

NG participated in the design of the study, supervised the laboratory work, and drafted the manuscript.

All authors read and approved the final manuscript.

## Pre-publication history

The pre-publication history for this paper can be accessed here:



## Figures and Tables

**Table 1:** Summarized patient data

Characteristics	Number	%
Number of patients	61	
Age of diagnosis (years)		
Range	31–72	
Median	47	
Histology		
Fibroadenoma	10	16.4
Hyperplastic adenosis/Fibroadenoma	10	16.4
Invasive ductal	31	50.8
Invasive lobular	4	6.6
Invasive ductal-lobular	6	9.8
Stage (breast carcinoma)		
I	6	14.6
II	24	58.5
III	8	19.5
IV	3	7.3
Estrogen receptors (only for breast carcinoma)		
Negative	22	53.7
Positive	19	46.3
Progesterone receptors (only for breast carcinoma)		
Negative	24	58.5
Positive	17	41.5
ErbB2 receptors (only for breast carcinoma)		
Negative	23	56.1
Positive	18	43.9

**Table 2:** Fra-1 expression in benign and malignant breast tissues by immunohistochemistry

Histological type	Fra-1 staining score*			
	
	Nuclear	n	cytoplasm	n
Benign	High	11	High	0
	Low	9	Low	3
	Negative	0	Negative	17
Carcinomas	High	30	High	14
	Low	11	Low	23
	Negative	0	Negative	4

* Fra-1 staining score was as follows: for nuclear staining: high, >75% positive cells and strong staining; low, <75% positive cells and weak staining; for cytoplasmic staining: high, strong staining; low, weak staining.

**Table 3:** Correlation of Fra-1 cytoplasmic expression with benign and malignancy

Tumor	Cytoplasm Fra-1 expression		*P *value
		
	Positive(%)	Negative (%)	
Benign	3 (15.0)	17 (85.0)	0.000
Malignancy*	37 (90.2)	4 (9.8)	

* The positive cytoplasmic immunohistochemical reactivity of Fra-1 in breast carcinomas includes strong and weak staining.

**Table 4:** Correlation of Fra-1 cytoplasmic expression with other prognostic factors

Characteristics	Cytoplasm Fra-1 expression		*P*
		
	Positive (%)	Negative (%)	
ER			
negative	17 (77.3)	5 (22.7)	1.00
positive	15 (78.9)	4 (21.9)	
PR			
negative	21 (87.5)	3 (12.5)	0.128
positive	11 (35.3)	6 (64.7)	
HER-2			
negative	18 (78.3)	5 (21.7)	1.00
positive	14 (77.8)	4 (22.2)	

* The positive cytoplasmic immunohistochemical reactivity of Fra-1 in breast carcinomas includes strong and weak staining. The ErbB2 status was classified as positive (2++ and 3+++) and negative (1+ and 0).
